# Microfluidics for protein interaction studies: current methods, challenges, and future perspectives

**DOI:** 10.1007/s00249-025-01763-x

**Published:** 2025-06-10

**Authors:** Matthias M. Schneider, Tuomas P. J. Knowles, Sandro Keller, Georg Krainer

**Affiliations:** 1 Centre for Misfolding Diseases, Yusuf Hamied Department of Chemistry, Lensfield Road, Cambridge, CB2 1EW UK; 2 Biophysics, Institute of Molecular Biosciences (IMB), NAWI Graz, University of Graz, Humboldtstr. 50/III, 8010 Graz, Austria; 3 Field of Excellence BioHealth, University of Graz, 8010 Graz, Austria; 4 BioTechMed-Graz, 8010 Graz, Austria

**Keywords:** Microfluidics, Protein interactions, Diffusion, Free-flow electrophoresis, Droplet microfluidics, Phase separation

## Abstract

Proteins are the key molecular players of life, carrying out their functions through interactions. Microfluidic technologies have emerged as powerful tools for studying protein interactions with exquisite sensitivity, resolution, and throughput. In this review, we highlight recent advances in microfluidic approaches for protein interaction studies. We first explore continuous-flow microfluidics, which utilize diffusion-based techniques and electrophoretic methods, before examining the role of droplet microfluidics in probing protein interactions. We provide an overview of the diverse applications of these technologies in biophysical research, drug discovery, and clinical diagnostics. We conclude with a discussion of the potential of microfluidics for driving future innovations and emerging opportunities.

## Introduction: the role of protein interactions and the rise of microfluidics

Proteins are the fundamental molecules of life (Crick 1970), functioning through interactions with other proteins and biomolecules to form complex networks that perform and regulate cellular functions. It is estimated that the human interactome consists of approximately 650,000 protein–protein interactions (Stumpf et al. 2008), which are fundamental to virtually all biological processes, from DNA replication (Nossal 1992) and transcription (Göös et al. 2022) to cellular signaling (Mayer 2006; Westermarck et al. 2013) and immune system responses (Goldberg 1952, 1953). Understanding these interactions is crucial for deciphering molecular mechanisms, mapping the interactome, and developing targeted therapies for diseases, such as cancer, neurodegeneration, and infectious diseases (Nada et al. 2024). Traditional techniques, including co-immunoprecipitation (Brymora et al. 2004; Free et al. 2009; Speth et al. 2014), yeast two-hybrid screening (Fields and Sternglanz 1994), mass spectrometry (Smits and Vermeulen 2016), nuclear magnetic resonance (NMR) spectroscopy (Wilkins et al. 1999; Jones et al. 1997), enzyme-based immunosorbent assay (ELISA) (Engvall 1980; Engvall and Perlmann 1972; Slutzki et al. 2012), and surface plasmon resonance (SPR) spectroscopy (Mullett et al. 2000; Beeg et al. 2019; Homola 2003; Visentin et al. 2016), have enabled significant advances in protein interaction studies but are often limited by high sample requirements, lack of real-time analysis (Phizicky and Fields 1995), or the need for immobilisation, which can distort native interactions (Whitesides 2006).

Microfluidics has emerged as a groundbreaking approach to overcome these limitations. Microfluidic technologies operate at the microscale, offering precise control over fluids (Beebe et al. 2002) and allowing researchers to study protein interactions with minimal reagent use and high sensitivity (Whitesides 2006). A key feature of microfluidic systems is their ability to exploit laminar flow, where fluids move in parallel streams without turbulence, facilitating diffusion-based mixing and precise manipulation of reaction conditions (Whitesides 2006). By enabling real-time analysis and compatibility with advanced detection tools such as single-molecule fluorescence, microfluidics provides unparalleled resolution in studying transient and weak interactions that are often challenging to detect using conventional bulk methods (Arter et al. 2020a). Furthermore, due to their slow rate of mixing under laminar flow conditions, there is no need for immobilisation of the analytes (Arter et al. 2020a). This minimises structural perturbations and allows assessment of protein behaviour in native-like environments under biologically relevant conditions (Schneider et al. 2023), which is especially beneficial for studying interactions within human-derived samples (Schneider et al. 2023), crowded cellular environments (Paguirigan and Beebe 2008), or those mediated by phase-separated biomolecular condensates (Arter et al. 2022).

The adaptability of microfluidic platforms also supports their use in diverse applications. High-throughput screening is one area where microfluidics excels, enabling rapid evaluation of binding kinetics and interaction dynamics (Dittrich and Manz 2006; Pihl et al. 2005). For example, the compartmentalisation of reactions in microdroplets facilitates the analysis of millions of potential binding events in a fraction of the time required by conventional methods. Furthermore, microfluidic systems are highly compatible with advanced detection technologies, such as time-resolved fluorescence spectroscopy and mass spectrometry (Rob and Wilson 2009), providing a holistic view of protein behaviour. This capability is particularly advantageous for studying transient interactions, such as those involving aggregation-prone or multi-component systems (Arter et al. 2020a).

However, the widespread adoption of microfluidics is not without challenges. The fabrication of microfluidic devices requires specialised expertise, and the lack of standardised protocols across laboratories can hinder reproducibility. The integration of user-friendly designs and modular systems is crucial for overcoming these barriers and ensuring accessibility to a broader scientific community.

In this review, we provide an in-depth exploration of microfluidic technologies and their application in protein interaction studies. Each approach offers unique advantages for specific applications. We examine the fundamental principles that make microfluidics a powerful tool, highlight its diverse applications, and discuss the challenges and opportunities that lie ahead in advancing protein interaction research. We do not aim at giving a concise description of all methods and applications, but rather to describe particularly prominent methods and to highlight case studies that give a sense of their utility in different areas of biophysical research.

## Continuous-flow microfluidics

Microfluidic methods for studying protein interactions can be broadly categorised into (i) continuous-flow techniques, in which the protein sample remains dissolved in one—typically aqueous—phase and (ii) droplet-based systems, in which proteins are encapsulated into smaller compartments or emulsions. The continuous-flow assays can be further separated into diffusion-based techniques and electrophoretic techniques. In the following, recent advances in these techniques are summarised.

### Diffusion-based techniques

Diffusion-based methods leverage on the well-understood movement of molecules in microfluidic systems. At low Reynolds numbers in a microfluidic channel, two adjacent streams only mix by diffusion-limited mass exchange, known as laminar flow (Müller et al. 2015). Therefore, these techniques allow for measurement of the diffusion coefficient. The diffusion coefficient is inversely proportional to the hydrodynamic radius via the Stokes–Einstein law (Einstein 1905). Upon binding to an interactor, the apparent hydrodynamic radius of a protein increases (Arter et al. 2020a). This size-increase not only gives qualitative information that the proteins interact, but via precise control of the concentration conditions, it becomes possible to assess both thermodynamic and kinetic parameters describing the protein interaction (Otzen et al. 2021). Diffusion-based techniques also allow for real-time monitoring of protein interactions in solution, offering a significant advantage over immobilisation-dependent methods (Arter et al. 2020a). The integration of sensitive fluorescence-based detection further enhances sensitivity, enabling the study of low-abundance proteins and weak interactions under native conditions.

**Fig. 1:**
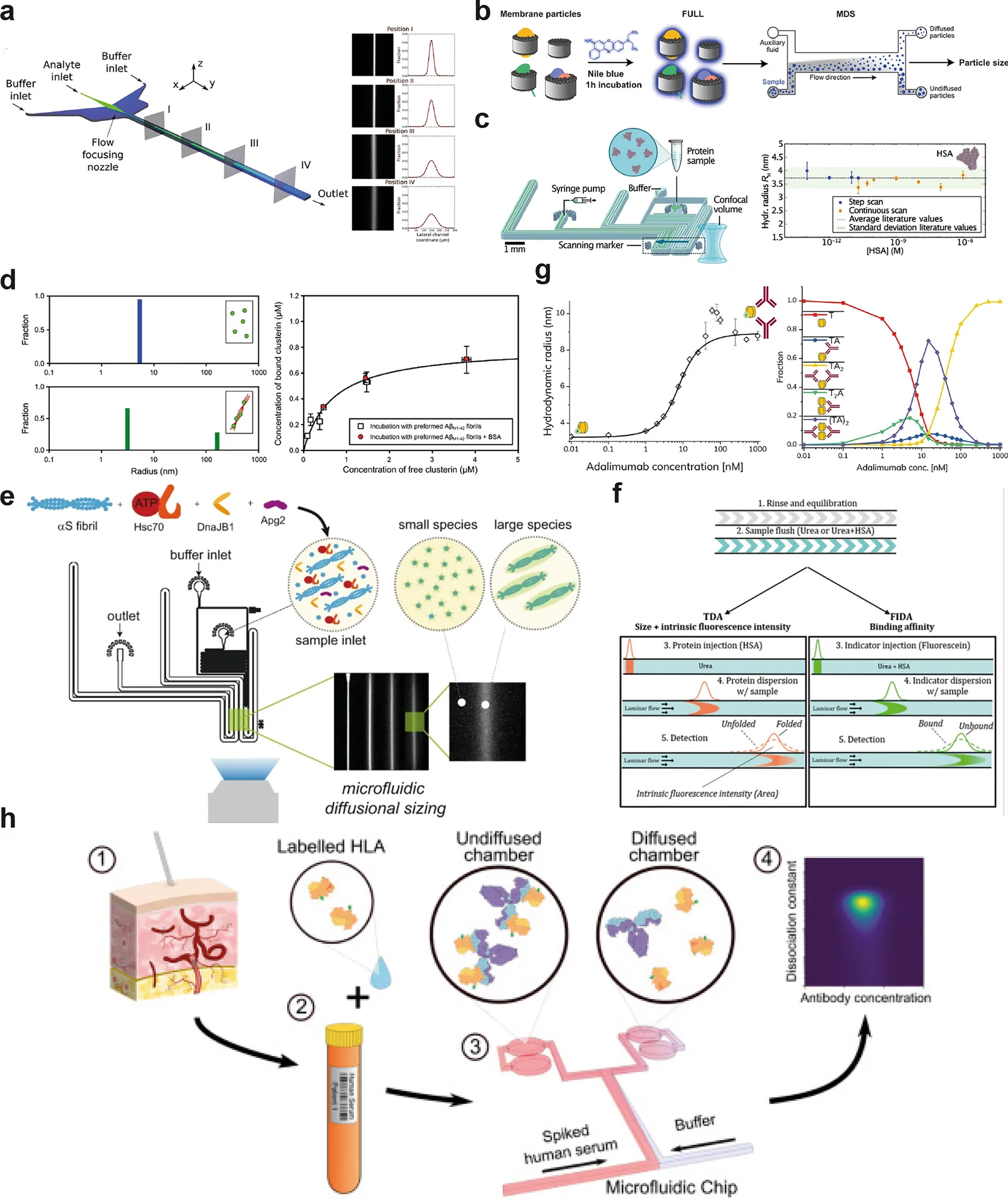
Diffusion-based methods to study protein interactions. **a** Principle of microfluidic diffusional sizing (MDS). A sample stream is introduced centred in a microfluidic channel, surrounded by auxiliary buffer. The diffusive broadening is traced at four different positions along the diffusion channel, corresponding to four diffusion times, for determination of the diffusion coefficient. Figure taken from (Arosio et al. 2016). **b** Workflow of FULL–MDS. Lipid nanodiscs are incubated with the fluorescence dye Nile blue, which spontaneously inserts into the lipid bilayer. This allows subsequent measurements by MDS. Figure taken from (Baron et al. 2023). **c** Single-molecule MDS (smMDS) relies on scanning a confocal volume across the microfluidic channel (left), allowing for accurate size determination down to 100 fM (right). Figure taken from (Krainer et al. 2024). **d** MDS can be used to distinguish clusterin that is unbound from clusterin that is bound to Aβ42 fibrils. Relative titration enables determination of binding affinities and binding stoichiometries. Figure taken from (Scheidt et al. 2019). **e** MDS can be used to follow the disaggregation kinetics of Hsc70 mediated disaggregation of α-synuclein (αSyn). Figure taken from (Schneider et al. 2021). **f** Working principle of Taylor dispersion analysis (TDA) and flow-induced dispersion analysis (FIDA). A sample is inserted under laminar flow into a microcapillary, and the diffusive dispersion of the fluorescence is measured. Figure taken from (Otzen et al. 2021). **g** Hydrodynamic radius of Alexa-labelled TNF-α as a function of the concentration of adalimumab, yielding a binding curve (left). This allows to simulate the fractions of complexes with different stoichiometries present in the reaction mixture (right). Figure taken from (Pedersen et al. 2021). **h** Working principle of microfluidic antibody affinity profiling (MAAP). Labelled antigen is incubated with antibodies in pristine human serum and subsequently subjected to an MDS platform. Titration of the relative concentrations allows assessment of antibody affinity and serum antibody concentration. Figure taken from (Schneider et al. 2023)

One prominent example of a diffusion-based technique is microfluidic diffusional sizing (MDS). In MDS, a protein sample stream flows through the centre of a microfluidic channel, flanked by auxiliary buffer streams (see Fig. 1a) (Arosio et al. 2016). Under laminar flow conditions, protein molecules can enter the surrounding buffer by diffusion only (Arosio et al. 2016). By measuring the extent of diffusive broadening at different positions along the channel—corresponding to varying diffusion times—the diffusion coefficient of the sample can be determined, allowing for the calculation of its hydrodynamic radius (Arosio et al. 2016). For the detection of protein complexes, in its initial version, MDS relies on fluorescent labelling with organic fluorophores (e.g., Alexa Fluor dyes) (Arosio et al. 2016), while later implementations also operate label-free, relying on detecting protein auto-fluorescence in the ultraviolet spectral region (Challa et al. 2018) and in situ labelling on the MDS chip (Yates et al. 2015). While such labelling methods are useful for proteins, lipids are particularly challenging to label. For such applications, fluorescent universal lipid labelling MDS (FULL–MDS) has been developed, relying on non-covalent labelling with fluorophores Nile blue and Nile red, which spontaneously partition into hydrophobic lipid bilayers (see Fig. 1b) (Baron et al. 2023). Recent advances allow for the integration of single-molecule detection (smMDS), enabling the detection of proteins down to 100 fM concentration (Fig. 1c) (Krainer et al. 2024). Using smMDS, it becomes possible to determine interaction affinities at single-molecule levels, resolving assembly states of high- and low-molecular-weight protein oligomers, and to size multiple protein species in complex aggregation mixtures (Krainer et al. 2024).

MDS has been applied to study diverse fundamental biophysical phenomena. A particular focus has been laid on interactions of ligands with amyloid fibrils, which are often associated with neurodegenerative diseases. For example, binding of a labelled nanobody to fibrillar α-synuclein (αSyn), which is related to Parkinson’s disease, demonstrated that MDS allows differentiating between fibril-bound and unbound nanobody molecules based on the significant difference in diffusion rate (Zhang et al. 2016). MDS has been used to differentiate up to three species in a reaction mixture (Schneider et al. 2021). Scheidt et al. demonstrated that relative titration of two interaction partners allows for the detection of both binding affinities and binding stoichiometries (Fig. 1d) (Scheidt et al. 2019). Measuring the interaction between the molecular chaperone clusterin and fibrillar amyloid-β 42 (Aβ42), which is related to Alzheimer’s disease, the authors demonstrated that the binding stoichiometries measured through the MDS approach agreed with independent results obtained by transmission electron microscopy (TEM) (Scheidt et al. 2019). This titration assay has since been applied to many other systems, including the interaction of the proinflammatory S100 A9 protein to Aβ42 (Pansieri et al. 2020), the molecular chaperone αB crystalline to αSyn (Scheidt et al. 2021), clinical antibodies to Aβ42 (Linse et al. 2020), designer peptides to Aβ42 (Sahtoe et al. 2024) and αSyn (Bismut et al. 2025), antibodies to DNA (Ausserwöger et al. 2023), and trigger factor to ribosomes (Herling et al. 2024), among others. Apart from interactions of ligands with amyloid, MDS has been applied to studying the oligomerisation of Hsp70 (Wright et al. 2018) and the aggregation of insulin (Saar et al. 2016), allowing for the real-time analysis of oligomerisation processes, enabling assessment of reaction kinetics. This has been used to study the disaggregation functionality of the Hsc70–DnaJB1–Apg2 chaperone system on fibrillar αSyn, providing detailed mechanistic insights into this reaction for the first time (Fig. 1e) (Schneider et al. 2021).

Another prominent diffusion-based technique is flow-induced dispersion analysis (FIDA) (Jensen and Østergaard 2010). FIDA relies on the principle of Taylor dispersion analysis (TDA). In TDA, a protein sample is injected into a silica capillary under laminar flow (Fig. 1f) (Taylor 1953). The protein sample is dispersed according to Fick’s 2nd law of diffusion (Taylor 1953), and fitting the dispersion curve allows determination of the diffusion coefficient and derived physical parameters (Otzen et al. 2021). Mixing of the fluorescent indicator (ligand) with the analyte of interest can occur either directly in the capillary or prior to sample loading (Otzen et al. 2021). As for MDS, titrating different analyte concentrations allows to assess binding affinities (Otzen et al. 2021).

FIDA has been used in a variety of settings to study protein interactions. For example, the interaction between the trimeric protein TNF-α and the antibody adalimumab was probed, demonstrating that FIDA can distinguish up to six different oligomerisation states that are simultaneously abundant in the reaction mixture (Fig. 1g) (Pedersen et al. 2021). This compares favourably with the three species that can be resolved by MDS. Further applications of FIDA include studying the oligomerisation of insulin (Jensen et al. 2014), the interaction of monomeric αSyn with phospholipid vesicles (Cholak et al. 2020), and interactions of monoclonal antibodies with oligomeric αSyn (Nielsen et al. 2024). Likewise, by separating the non-diffusive from the diffusive signals in the analysis through transient incomplete separation, it becomes possible to study the aggregation behaviour of αSyn, as well as their thermodynamic stability based on urea-induced depolymerisation (Farzadfard et al. 2024). Recently, continuous titration based spectral related intensity change (cSPRING) was developed, which allows rapid titration of a ligand into the capillary and, thus, rapid screening of the affinity (Willmer et al. 2025*).*

Moreover, recent advances have demonstrated the applicability of FIDA to study phase separation of proteins. The Buell group has pioneered a technique, known as Capflex (Stender et al. 2021), which relies on detecting droplets as individual spikes based on the fluorescent signal of biomolecular condensates. From these fluorescent signals, key parameters describing phase separation can be readily assessed, including the protein concentration in the dilute phase, the droplet size, the effect of inhibitors, and the kinetics of droplet formation (Stender et al. 2021). A further development of this method is Taylor dispersion-induced phase separation (TDIPS) (Norrild et al. 2024), which relies on the comparison of the assembly state (monomeric vs. cluster) of phase-separating proteins under various salt conditions or inhibitor concentrations (Norrild et al. 2024).

In a recent study, Willmer et al. demonstrate that FIDA also allows the detection of protein interactions under non-equilibrium conditions, known as C-Jump (Willmer et al. 2025). Through mixing in the capillary, it becomes possible to detect the protein complexes before equilibrium is reached, yielding information on the kinetics of protein interactions and, therefore, better emulates the native conditions under which protein interactions take place (Willmer et al. 2025).

Both MDS and FIDA have been widely applied in immunoassays. For example, with MDS, the abundance of antibody–antigen interactions in full blood were determined (Hatch et al. 2001). Furthermore, MDS was used to measure alloantibody interactions with human leukocyte antigen (HLA) isoforms in human serum—an important biomarker for organ transplantation (Fig. 1h) (Schneider et al. 2023). This immunoassay has been widely applied for characterising the interaction of serum antibodies with the peanut allergen Ara h2 (Emmenegger et al. 2024) and the receptor binding domain of the spike protein of SARS-CoV-2 (Schneider et al. 2022), and competition assays were developed to determine the neutralisation potential (Fiedler et al. 2021) and the cross-reactivity (Denninger et al. 2022; Fiedler et al. 2021) of these serum antibodies. Likewise, FIDA is also applicable for measurements in complex human liquids such as serum. It has been used to study anti-dsDNA antibodies in plasma from patients suffering from systemic lupus erythematosus (Poulsen et al. 2016), anti-albumin antibodies in plasma (Poulsen et al. 2015), monoclonal antibodies in fermentation broth (Pedersen et al. 2020), and signalling lipid sphingosine 1-phosphate (S1P) with its carrier molecules human serum albumin (HSA) and high-density lipoprotein (HDL), a marker for sepsis (Seidita et al. 2024).

### Electrophoretic methods

Electrophoretic methods are widespread in biomolecular research and are usually employed to separate and analyse biomolecules on a solid support matrix, such as an agarose or polyacrylamide gel (Minden et al. 2009; Aaij and Borst 1972; Johansson 1972). These methods usually require large sample quantities, and measurement times usually range between minutes and hours (Johansson 1972). Separation usually occurs based on the charge/size ratio of the proteins, and often it is difficult to differentiate between proteins with similar molecular weight (Arter et al. 2020a). To achieve such sensitivity, immunofluorescence detection is usually required, foremost through Western blotting (Kurien and Scofield 2006).

To tackle these limitations, electrophoresis has been integrated in microfluidic technologies. Since mixing of liquids on the microscale is limited by diffusion, there is no need for separation through a solid support matrix (Arter et al. 2020a). By applying an electric field across a microfluidic channel, protein complexes are separated based on their charge/mass ratios, rendering quantitative information such as the electrophoretic mobility (Arter et al. 2020a). Separation and fractionation of biomolecules allow for the characterisation of weak and transient biomolecular interactions without calibration (Arter et al. 2020a). Moreover, the ability to perform these analyses in a miniaturised format reduces reagent consumption and enables rapid throughput.

**Fig. 2:**
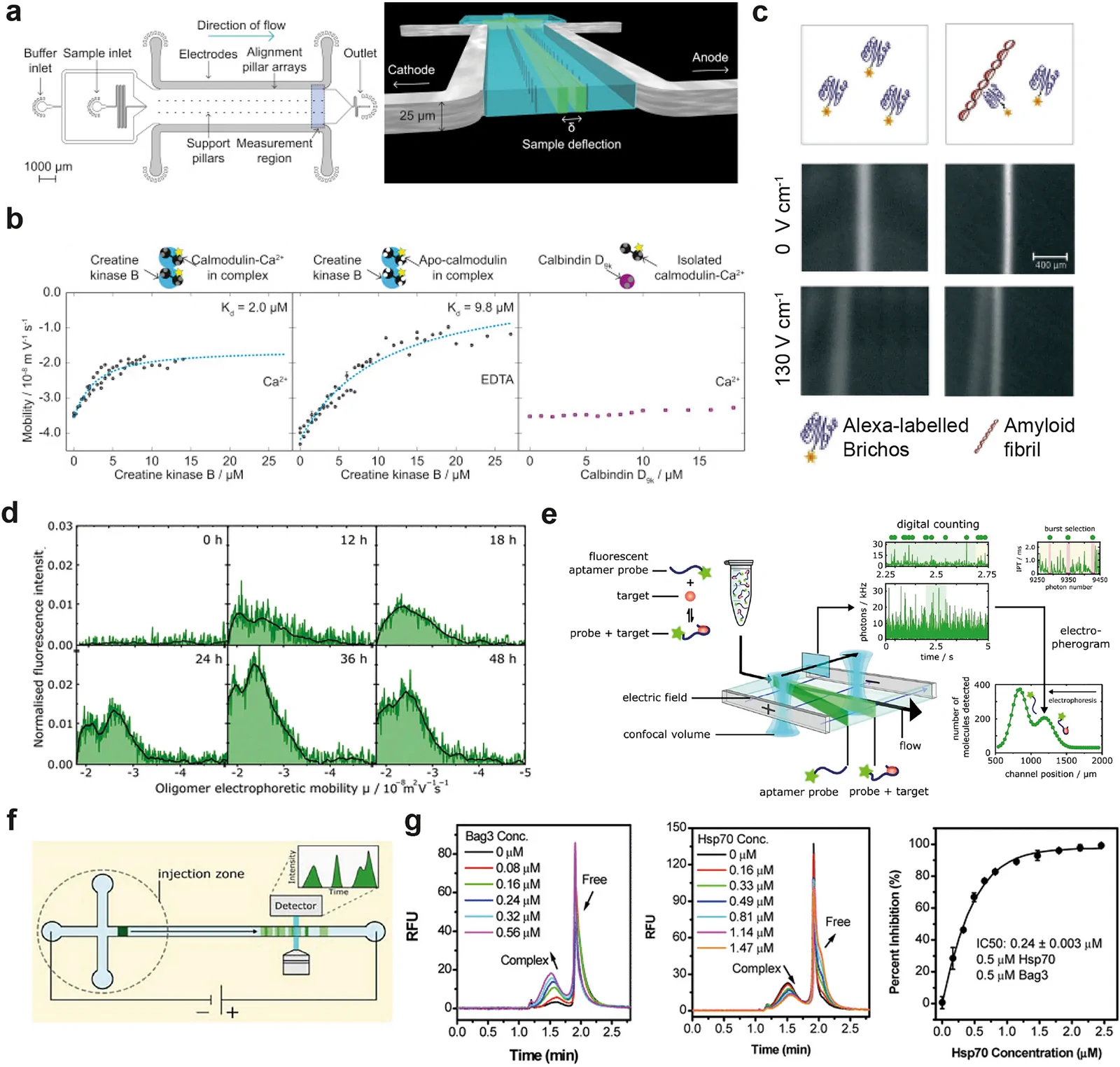
Electrophoresis-based microfluidic techniques to study protein interactions. **a** Design of a microfluidic free-flow electrophoresis device, with solid electrodes in grey (left). The deflection is measured at the grey shaded area. Upon application of an electrophoretic current, the sample is deflected towards the anode (right). Figure taken from (Herling et al. 2016). **b** Change of the electrophoretic mobility of calmodulin as a function of creatine kinase B concentration in presence (left) and absence (middle) of Ca^2+^ ions. The electrophoretic mobility of calmodulin in absence of creatine kinase B remains unchanged (right). Figure taken from (Herling et al. 2016). **c** Electrophoretic mobility of Alexa-488 labelled Brichos in free solution (top) and when bound to Aβ42 fibrils (bottom). Figure taken from (Saar et al. 2017). **d** Electropherograms of the oligomeric α-synuclein (αSyn) populations at different aggregation times, revealing shifts in the oligomers present at different stages of the aggregation reaction. Figure taken from (Arter et al. 2020b). **e** Working principle of digital immunosensor assay (DigitISA). A fluorescently labelled aptamer is indicated with the target, and target bound aptamer is separated from free aptamer by electrophoresis by single molecule counting. Figure taken from (Krainer et al. 2023). **f** Principle of microcapillary electrophoresis. Fluorescent protein mixture is introduced into a microcapillary, along which an electric field is applied, leading to separation of different protein fractions. Figure taken from (Arter et al. 2020a). **g** Electropherograms of 0.5 μM Hsp70 with varying concentration of Bag3 and of 0.5 μM Bag3 (left) with various concentration of Hsp70 (middle). The complex formation was inhibited with increasing Hsp70 concentration (right). Figure taken from (Rauch et al. 2013)

There are two main electrophoretic techniques: free-flow electrophoresis (μFFE) and micro-capillary electrophoresis (MCE). μFFE is based on flowing a protein sample stream in the centre of a microfluidic chip surrounded by auxiliary buffer (Fig. 2a) (Herling et al. 2013). On both sides, parallel to the flow direction of the microfluidic chamber, an electrode is in direct contact with the auxiliary buffer, allowing for the application of an electric field perpendicular to the flow direction (Herling et al. 2013). These electrodes can be solid, made of an InBiSn alloy (Herling et al. 2013), or liquid, made of 3 M KCl (Saar et al. 2017; Saar et al. 2018). The deflection of protein molecules in the electric field can be used to quantify their electrophoretic mobility (Herling et al. 2013), which is possible, because the device operates fully in solution and, hence, the proteins remain in their native structure (Arter et al. 2020a). While traditional FFE implementations suffer from Joule–heating (Tarnopolsky et al. 1993; Mahmud et al. 2023), the high surface area-to-volume ratio of the microscale channels facilitates rapid heat transfer, minimising the detrimental effect (Saar et al. 2018). This assay can be used to study protein interactions that would not be accessible through diffusion assays, because the increase in complex size would be too small. For example, it is possible to differentiate between calcium-bound and -unbound states of the calcium ion binding protein calbindin D_9k_ in free solution (Herling et al. 2015), and to track the calcium dependence of the interaction between calmodulin and creatine kinase B (Fig. 2b) (Herling et al. 2016). Furthermore, μFFE can be used to detect binding of ligands to amyloid fibrils, such as proSPc Brichos to fibrillar Aβ42 (Fig. 2c) (Saar et al. 2017). While the protein sample is usually fluorescently labelled with an Alexa dye, μFFE can also be operated by detecting autofluorescence of the protein, as outlined in a study characterising the oligomerisation potential of the molecular chaperone αB crystalline (Wright et al. 2019).

Arter et al. showed that μFFE can be used to fractionate samples based on their different electrophoretic properties (Arter et al. 2018). For this purpose, they used a labelled aptamer binding to a small oligonucleotide that was linked to a biotin marker (Arter et al. 2018). Aptamer molecules bound to the biotin marker were deflected to the opposite electrode, allowing for accurate quantification of the biotin concentration (Arter et al. 2018). Furthermore, this technique is very suitable to resolve heterogeneities. For example, it becomes possible to characterise transient aggregation intermediates, known as oligomers, of αSyn during the aggregation reaction course (Fig. 2d) (Arter et al. 2020b). Measuring the oligomer mass concentration during the time course of αSyn aggregation dependent on the initial seed concentration provided direct experimental evidence for secondary nucleation as the main mechanism for generating αSyn oligomers (Xu et al. 2024). Likewise, μFFE has been used to characterise the binding of oligomeric αSyn to lipid vesicles (Šneiderienė et al. 2024) and the off-target binding of antibodies to DNA (Herling et al. 2023). Moreover, μFFE has also proven useful in the field of biomolecular phase separation. Loading biomolecular condensates into the μFFE setup, the surface-charge of these condensates can be measured (Welsh et al. 2022). Using this technique, it was possible to show that the surface charge is a key modulator of condensate stability (Welsh et al. 2022). Recently, integration of μFFE with confocal fluorescence detection led to a technique known as digital immunosensor assay (DigitISA, Fig. 2e) (Krainer et al. 2023). DigitISA allows for direct quantification of the number and concentration of a wide range of protein targets binding to a labelled aptamer (Krainer et al. 2023). As such, DigitISA holds promise for applications in protein biosensing and clinical diagnostics.

In MCE, the sample is introduced into a microcapillary prior to application of a parallel electric field, causing electrophoretic separation of different protein bands inside the microcapillary (Fig. 2f) (Ouimet et al. 2017). For example, MCE was applied to characterise the interaction of Hsp70 with its co-chaperone Bag3 (Rauch et al. 2013), an important interaction in the cellular stress response (Meriin et al. 2018), which also stabilises certain oncogens (Chiappetta et al. 2007). Rauch et al. demonstrated that, using MCE, it becomes possible to differentiate between fluorescently labelled Hsp70 that is bound and unbound to Bag3 (Fig. 2g) (Rauch et al. 2013). The authors used this feature to evaluate the efficiency of different drug candidates that act as inhibitors for this recognition (Rauch et al. 2013). In another study, it was possible to differentiate several hundred aggregation states of Aβ42 in the microcapillary (Picou et al. 2012). MCE is also particularly useful for studying post-translational modifications (PTMs) that alter protein charge. For example, MCE offered a deeper understanding of modulators for tau phosphorylation (Nehmé et al. 2015).

## Droplet-based systems

**Fig. 3:**
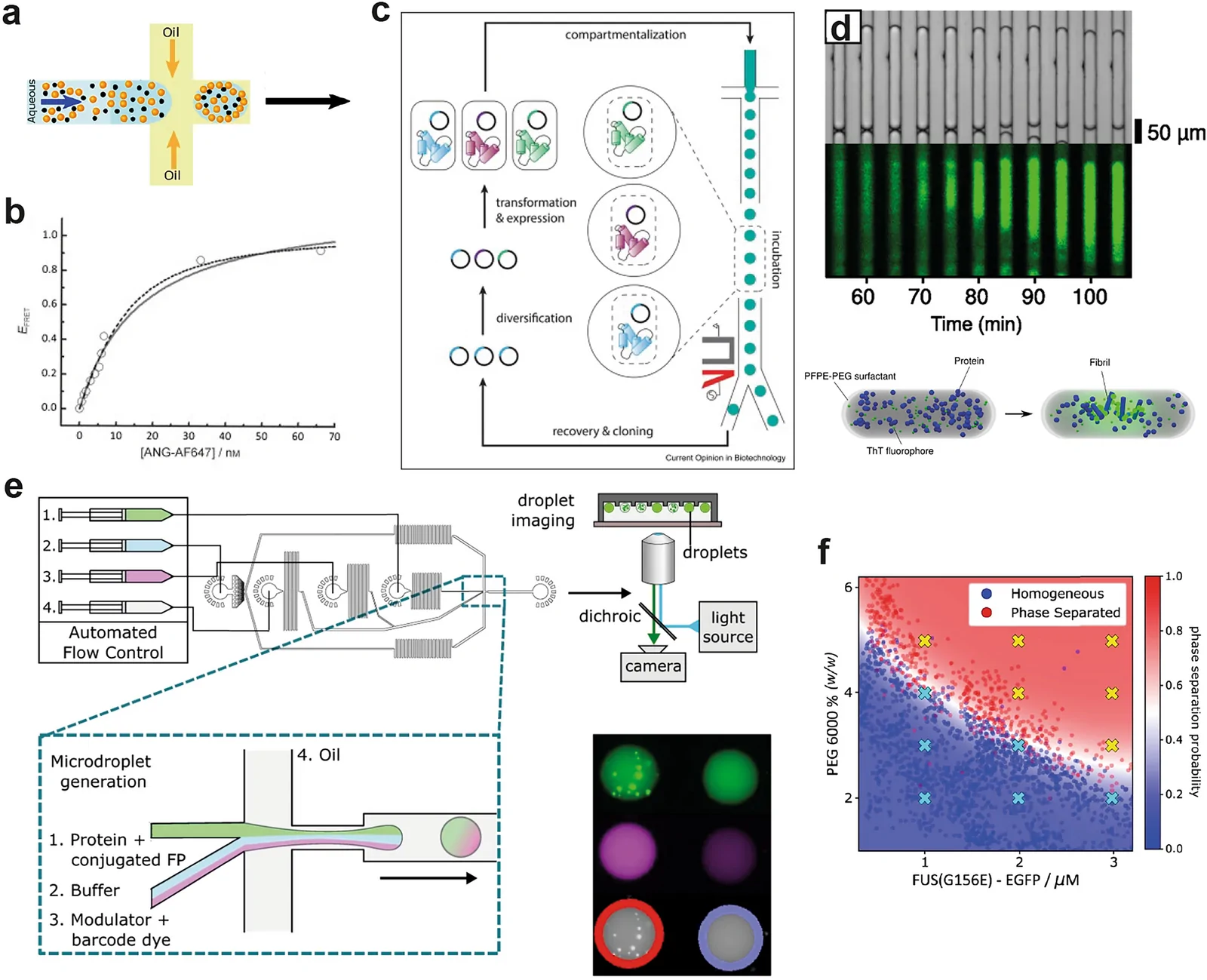
Droplet-based methods for assessing protein interactions. **a** Principle of droplet generation. In a microfluidic chip, an aqueous protein suspension merges with a surrounding oil phase, leading to droplet formation. Figure taken from (Toprakcioglu and Knowles 2021). **b** Binding curve for the interaction of angiogenin (ANG) with an anti-ANG antibody based on the fluorescence emission in the FRET channel. Taken from (Srisa-Art et al. 2009). **c** Microfluidic work-flow for enzyme screening. Bacteria that are transformed with a genetic enzyme library are encapsulated in microdroplets. These microdroplets contain an enzyme substrate that turns fluorescent upon reaction. The droplets are then subjected to a microfluidic sorting device, leading to selection of droplets containing active enzymes. Figure taken from (Jain et al. 2024). **d** Propagation of amyloid formation of insulin in microdroplets in brightfield (top) and fluorescence (bottom) channel. Figure taken from (Knowles et al. 2011). **e** Principle of PhaseScan. Droplets are generated in a flow-centred microfluidic device. At the injection junction, different solutions containing phase-separating protein of interest, modulators, and buffer mix under laminar flow, before being encapsulated by an oil phase. The resulting droplets are captured in wells and imaged using fluorescence microscopy. The resulting images (bottom right) allow to distinguish between phase-separated (left) and non-phase-separated (right) states in two fluoresce channels, Alexa 488 (top) and Alexa647 (middle), allowing classification of the phase-separation state (bottom). Figure taken from (Arter et al. 2022). **f** Phase diagram for the interaction of FUS with PEG. Taken from (Arter et al. 2022)

In contrast to the aqueous-phase microfluidic methodologies discussed above, droplet microfluidics relies on the co-existence of two phases, usually an oil phase and an aqueous phase (Fig. 3a) (Theberge et al. 2010). By flowing an aqueous phase into an oil phase, emulsions or droplets with a narrow size distribution can be achieved (Umbanhowar et al. 2000). This allows encapsulation of proteins and reagents in picoliter-sized droplets (Arter et al. 2020a). Readouts are usually based on a fluorescence marker that distinguishes between droplets in which an interaction occurs and droplets without interaction. For example, Srisa-Art et al. used droplet microfluidics to demonstrate the interaction between an angiogenin (ANG) and an angiogenin-specific antibody (Fig. 3b) (Srisa-Art et al. 2009). They achieved determination of the interaction with Förster resonance energy transfer (FRET) microscopy by labelling the two interaction partners with a set of fluorophores that can act as a FRET pair (Srisa-Art et al. 2009). From the shift in the emission wavelength, it becomes possible to determine whether the antibody is bound to its antigen (Srisa-Art et al. 2009). Alternatively, fluorescence polarisation readouts have been reported, such as in a study of the interaction between ANG and an anti-ANG antibody (Choi et al. 2012). While unbound, fluorescently labelled ANG can freely rotate in solution during its fluorescence lifetime, causing emission of depolarised light, ANG in complex with its antibody is restricted in its rotation, leading to the emission of polarised light (Choi et al. 2012). Alternatively, detection can rely on protein substrates that turn fluorescent upon interaction, a tool that is widely used in enzyme discovery (Jain et al. 2024).

Using such assays, it becomes feasible to track millions of parallel reactions within a single experiment, facilitating high-throughput screening of protein interactions (Agresti et al. 2010), with typical frequencies of ~ 2000 Hz (Baret et al. 2009). A field in which this feature has been explored is the directed evolution of enzymes for detecting the interactions between an enzyme and its substrates. In enzyme development, iterative cycles of mutagenesis and subsequent activity screening and selection are employed to improve the catalytic activities (Zeymer and Hilvert 2018). While originally relying on microplate-based assays, droplet microfluidic platforms have emerged as an alternative screening method (Fig. 3c) (Jain et al. 2024). This has been demonstrated on a wide range of enzymes, including horse radish peroxidase (Agresti et al. 2010), class I aldolases (Obexer et al. 2016), and a manganese-independent α-L-threofuranosyl nucleic acid (TNA) polymerase (Larsen, et al. 2016), to name but a few.

Droplet-based systems have been instrumental in studying protein aggregation. For instance, droplets containing Aβ42 allowed researchers to follow primary nucleation, a process that is not commonly observable under bulk experimental conditions, where aggregation is dominated by secondary nucleation (Cohen, et al. 2013). Confinement of monomeric insulin into a microdroplet allowed to study single nucleation events in each droplet, and to trace the subsequent spatial and temporal evolution of the aggregates (Knowles et al. 2011). Deformation of droplets provided the finding that the aggregation propagates along the fibril surface, yielding experimental evidence for the importance of secondary nucleation in amyloid formation (Fig. 3d) (Knowles et al. 2011). Similarly, an assay was developed that could absolutely quantify the self-replicating units of fibrillar insulin, propagons, through digital counting (Pfammatter et al. 2017). While these examples all rely on fluorescently labelled protein aggregates, Toprakcioglu et al. showed in an extensive study that the aggregation of lysozyme, reconstituted silk protein, and β-lactoglobulin can be followed by measuring intrinsic tryptophane fluorescence (Toprakcioglu et al. 2019).

Droplet microfluidics has also opened new avenues in the study of biomolecular condensates, which are liquid-like assemblies formed by phase separation of proteins, nucleic acids, and other biomolecules (Banani et al. 2017). These condensates regulate essential cellular processes, such as transcription and stress response (Banani et al. 2017). To characterise the formation of biomolecular condensates, phase diagrams are commonly recorded, which display the dependency of the phase-separation behaviour based on parameters, such as relative concentrations of the biomolecular components, the temperature, crowding, or pH, among others (Gao et al. 2022; Alberti and Dormann 2019). In addition, shifts of the phase-separation behaviour are often accessed when screening phase-separation inhibitors (Dao 2022). However, recording phase diagrams with high accuracy and high throughput using standard laboratory techniques remains cumbersome (Alberti et al. 2019). Therefore, a droplet microfluidic approach was developed that allows rapid, combinatorial generation microcompartments that vary in their composition, known as PhaseScan (Fig. 3e) (Arter et al. 2022). The composition of the condensates can be accurately quantified using the fluorescence intensity of the constituent components (Arter et al. 2022). For phase separation of the protein fused in sarcoma (FUS), triggered with polyethylene glycol (PEG), it was possible to generate ~ 2600 droplets on a minute time scale, which were then classified as either phase-separated or non-phase-separated, yielding an accurate phase diagram for this system (Fig. 3f) (Arter et al. 2022). This approach has been widely used for a range of phase-separating systems, including FUS–RNA (Arter et al. 2022), FUS-salt (Arter et al. 2022), G3BP1–RNA (Arter et al. 2022), G3BP1–PEG (Arter et al. 2022), antimicrobial peptides with RNA (Sneideris, et al. 2023), antibodies with DNA (Ausserwöger et al. 2023), insulin-pH (Ausserwöger, et al. 2024), poly-A RNA with PEG (Erkamp, et al. 2023), and temperature-dependent measurements of G3BP1 phase separation (Fischer, et al. 2024), among others. Importantly, PhaseScan enables the recording of multidimensional phase diagrams, which is crucial for evaluating phase behaviour across a broader chemical space.

In addition to studying protein interactions and protein self-assembly for research purposes, microfluidic droplet assays are particularly valuable in drug discovery, enabling rapid testing of small molecules or antibodies against target proteins (Hong et al. 2009). For example, Debs et al. generated microdroplets containing hybridoma cells producing antibodies against angiotensin converting enzyme 1 (ACE-1) along with the ACE-1 enzyme (Debs et al. 2012), which is linked to hypertension and congestive heart failure (Zaman et al. 2002). After 6 h of antibody production, these droplets were fused to other microdroplets containing a substrate peptide of the ACE-1 linked to a tag which becomes fluorescent upon hydrolysis (Debs et al. 2012). This allowed sorting of fluorescent and non-fluorescent droplets, in which ACE-1 inhibiting antibodies could be identified (Debs et al. 2012). In another study, encapsulation of single cells into droplets has been used to test the effectiveness of the cancer drug temsirolimus against the proliferation of *Saccharomyces cerevisiae* cells (Kasim et al. 2021). Likewise, encapsulation of live cells from pancreatic cancers has been used to screen their reactivity against different drug combinations (Eduati, et al. 2018), and for testing the reactivity of cancer spheroids to chemotherapeutics (Parent et al. 2023).

In synthetic biology, microfluidic systems are being utilized to engineer artificial protein networks and generating artificial cells for mimicking cellular processes in a microdroplet (Gach et al. 2017; Ngocho et al. 2024). For instance, Kapsner et al. demonstrated that it is possible to study DNA transcription in a microdroplet to simulate a natural cellular environment (Kapsner and Simmel 2015). This study underlined the importance of the relative abundance of template DNA and polymerase for transcription efficiency, shedding light on the interaction between DNA polymerases and their templates. Furthermore, microfluidic systems have been developed to encapsulate lipid bilayers in microdroplets (Stachowiak et al. 2008), shedding light on the integration of membrane proteins into lipid bilayers (Tonooka et al. 2014) and their involvement in membrane transport (Tonooka et al. 2014; Czekalska et al. 2015; Soga et al. 2015) and cellular communication (Adamala et al. 2016). Furthermore, droplet-based systems have enabled the testing of modular protein circuits that mimic natural signalling pathways (Shang and Zhao 2021).

## Integration of techniques

One of the key strengths of microfluidic systems is their ability to integrate multiple analytical approaches into a single platform and to be coupled to other analytical methods. For example, diffusion-based sizing and electrophoretic separations have been coupled to obtain complementary data on protein size and charge (Scheidt et al. 2020). Scheidt et al. demonstrated that coupling such a chip design to size exclusion chromatography allows one to study the self-assembly behaviour of BSA, resolving the size, electrophoretic mobility, and surface charge in real time from each elution fraction coming off the chromatography system (Scheidt et al. 2020). Similarly, size exclusion chromatography was coupled to a microfluidic spray device, which lead to deposition of protein analytes on a quartz crystal microbalance for gravimetric quantification (Kartanas et al. 2021).

One of the most important integrations of microfluidic platforms for protein interaction analysis is with advanced detection systems. An important field is the integration of microfluidic techniques with single-molecule detection. For example, single-molecule MDS (smMDS), combining MDS with confocal microscopy, has allowed the sizing of proteins in the femtomolar concentration range and affinity determination of binary protein interactions with single-molecule resolution (Krainer et al. 2024). Furthermore, it became possible to resolve the heterogeneity of oligomer populations and to analyse protein species present in an aggregation mixture (Krainer et al. 2024). In another example, integration of μFFE with confocal microscopy in platform termed DigitISA enables digital counting of protein targets bound to specific, fluorescently labelled aptamers (Krainer et al. 2023). Such techniques are important stepping stones to advance microfluidic technologies for clinical applications. In addition, techniques based on single-molecule FRET (smFRET) have been implemented. For analysing smFRET, it is necessary to dilute the analytes of interest to low concentrations (Zijlstra et al. 2017). However, dilution effects often lead to dissociation of the complexes. Using a microfluidic dilution device, it becomes possible to generate the protein complexes at higher concentrations and dilute them by fivefold within milliseconds, allowing one not only to maintain the protein complex but also to track the dissociation kinetics upon dilution based on the FRET signal (Zijlstra et al. 2017). A study by Yang et al. demonstrated a microfluidic mixing device that enabled measurements of the association kinetics of two proteins, the intrinsically disordered activation domain of the p160 transcriptional coactivator (ACTR) and the nuclear coactivator binding domain (NCBD) of CREB-binding protein (Yang, et al. 2023). Upon binding, ACTR undergoes a conformational change. Labelling ACTR with a FRET-pair allows real-time observation of the association kinetics for this binding reaction in individual droplets on a single molecule level (Yang, et al. 2023). Likewise, using a passive microfluidic mixing device, it became possible to characterise scarcely populated folding intermediates under non-equilibrium conditions in real time (Dingfelder et al. 2017). The same technique was used for studying the dissociation kinetics of dimeric Hsp90 (Hellenkamp et al. 2018).

While most microfluidic methods rely on fluorescence detection, advances have been made to couple microfluidic devices to electron–spray (ESI–MS). For this purpose, a microfluidic spray device was developed (Ramsey and Ramsey 1997). Coupling a microfluidic chip to liquid chromatography and a spray device allows for separation of different fractions of a proteolytic digest sample (Mellors et al. 2013). This has been used to identify glycosylation sites of a monoclonal antibody (Mellors et al. 2013). Similar integration of droplet microfluidics with electrospray MS allowed for the characterisation of the droplet composition, with angiotensin as model compound (Zhu and Fang 2010). Similar integration of microfluidics with MALDI–MS allowed the analysis of a trypsin digest of proteins (Easley et al. 2009).

## Challenges in microfluidic protein interaction studies

Protein interactions are a central focus in biophysics and the life sciences more generally, and microfluidic techniques offer significant advantages over conventional methods for their characterisation. These advantages include minimal sample consumption, the ability to study protein behaviour under native physiological conditions, automation, and high-throughput analysis. Nevertheless, application of microfluidic techniques as tools for characterising protein interactions has remained limited to a few expert labs worldwide. In the following, we identify three main challenges which, in our opinion, hinder the more widespread application of microfluidics in protein interaction research and propose strategies to overcome these limitations.

First, the fabrication of microfluidic devices is complex, commonly relying on soft lithography approaches (Qin et al. 2010). These fabrication steps require specialised infrastructure, such as clean rooms, expensive equipment including plasma ovens, and specialist training. Furthermore, these soft lithography procedures are based on specialised materials, such as SU8 photoresists and polydimethylsiloxane (PDMS) polymers, which are toxic and expensive. Therefore, efforts should be directed towards the production of standardised, commercial chips. In addition, especially for application of microfluidic techniques in the context of clinical diagnostics (Schneider et al. 2023), customer-friendly bench-top instruments that allow rapid and standardised measurement of protein interactions are essential and would significantly reduce the costs associated with microfluidic methods.

Second, lack of standardised chip design and protocols for operating microfluidic procedures limits reproducibility across different laboratories (Pattanayak et al. 2021). This complicates comparisons between studies and slows the rapid development of guidelines for the optimal operation of microfluidic devices. While strategies to improve these limitations are discussed and addressed (Reyes et al. 2021), open-access sharing of protocols and chip designs would massively accelerate such efforts.

Third, operation of microfluidic devices and analysis of microfluidic data requires interdisciplinary knowledge from engineering, biology, statistics, and programming (Müller et al. 2015). This limits the application of microfluidic assays in protein interaction research, as not every biology lab employs experts in all these fields. Therefore, educational efforts would enable more labs to access the wide opportunities provided by microfluidic studies. In addition, foundation of interdisciplinary consortia that enable access of biologists to microfluidic techniques would provide access to expertise in microfluidics to a wider biological community.

## Conclusion, future directions, and emerging opportunities

Protein interactions underlie any biological functionality, and understanding protein interactions provides deep insights into the basic functions of life and an opportunity for drug discovery (Andrei et al. 2017). Microfluidic methods offer a unique approach to study such interactions, as they allow fast, real-time measurements of protein interactions in solution with no need of surface immobilisation or extensive calibration. The ability to perform high-resolution, real-time analyses with minimal sample requirements makes microfluidics an indispensable tool for advancing protein interaction research.

The field of microfluidic-based protein interaction studies is rapidly expanding, encompassing a wide range of emerging applications. One emerging field is research on biomolecular condensates. In that context, microfluidic techniques can be employed for the rapid characterisation of phase diagrams, on platforms such as PhaseScan (Arter et al. 2022). Furthermore, it becomes possible to determine physical properties of molecular condensates, such as surface charge (Welsh et al. 2022) or condensate size on a single-condensate level (Krainer et al. 2024). In addition, diffusion-based microfluidic techniques allow for the characterisation of soluble clusters that are precursors in the process of condensate formation (Ausserwöger et al. 2023).

Another emerging field in which microfluidic methods are employed is the study of membrane proteins. Membrane proteins are involved in cellular communication (Schumann 2022) and import (Pfanner et al. 1994), making them important targets for gaining deeper understanding into cellular function (Hedin et al. 2011), but also for assessing the role of membrane protein misfolding in diseases (Sanders and Myers 2004). For instance, MDS has been used to confirm the successful extraction of the bacterial potassium ion channel KvAP into polymer-encapsulated lipid-bilayer nanodiscs (Danielczak et al. 2022) and to study the charge-dependent interactions of α-synuclein and the peptide adrenocorticotropic hormone (ACTH) with anionic lipids embedded within such nanodiscs (Glueck et al. 2022). Droplet microfluidic methods have offered unique advantages for characterising interactions to membrane transport (Tonooka et al. 2014), and for the reconstitution of synthetic cells (Adamala et al. 2016). Furthermore, FULL–MDS allow flexible characterisation of any protein of interest to membrane proteins in lipid nanodiscs (Baron et al. 2023). There is vast potential for microfluidic approaches to study interactions of membrane proteins.

One of the most rapidly emerging fields in microfluidic protein interaction research are clinical and diagnostic screening. In an ageing society, and with many age-related diseases (Knowles et al. 2014), and with an increasing prevalence of cancers, it becomes increasingly important to recognise diseases early, in order to reduce their socio-economic burden (Chen-Plotkin, et al. 2018; Rasmussen and Langerman 2019; Shi et al. 2018). For such purposes, microfluidic techniques are ideally suited, as they allow detection of lower analyte concentrations compared to traditional technique, making microfluidics suitable for screening biomarkers in human fluids such as blood. Furthermore, microfluidics can have widespread applications in the validation of biomarkers (Tarasow et al. 2011; Nahavandi et al. 2014).

In summary, this review has shown that there hardly exist any conceptual limitations to applying microfluidics to study protein interactions, as the right technique can be found for virtually any research question. Mitigating limitations to the applicability of microfluidic systems in biological research will be important for unravelling the full potential of microfluidics in biophysical research and beyond.

## Data Availability

We do not believe that a data availability statement is applicable for a review article, as we did not include any actual data.
